# Middle Eastern Expert Opinion: Strategies for Successful Antifungal Stewardship Program Implementation in Invasive Fungal Infections

**DOI:** 10.7759/cureus.61127

**Published:** 2024-05-26

**Authors:** Jameela Alsalman, Abdulhakeem Althaqafi, Ahmad Alsaeed, Ahmad Subhi, Ahmed F Mady, Ayman AlHejazi, Bassam Francis, Hanan H Alturkistani, Mouhab Ayas, Montaser Bilbisi, Sondus Alsharidah

**Affiliations:** 1 Medicine, Arabian Gulf University, Manama, BHR; 2 College of Medicine, King Saud Bin Abdulaziz University for Health Sciences, Jeddah, SAU; 3 Infectious Diseases, King Abdullah International Medical Research Center, Jeddah, SAU; 4 Department of Medicine/Infectious Diseases, King Abdulaziz Medical City, Ministry of National Guard Health Affairs, Jeddah, SAU; 5 Adult Hematology and Stem Cell Transplantation, Princess Noorah Oncology Center, King Abdulaziz Medical City, Ministry of the National Guard Health Affairs, Jeddah, SAU; 6 Adult Infectious Diseases, Department of Medicine, Al-Qassimi Hospital, Emirates Health Services, Sharjah, ARE; 7 Critical Care Medicine, King Saud Medical City, Riyadh, SAU; 8 Department of Oncology, King Abdulaziz Medical City Riyadh, Riyadh, SAU; 9 College of Medicine, King Saud Bin Abdulaziz University for Health Sciences, Riyadh, SAU; 10 Hematology, Hematology and Bone Marrow Transplant Center, Baghdad, IRQ; 11 Transplant Infectious Diseases, King Abdullah Medical City, Makkah, SAU; 12 Department of Pediatric Hematology/Oncology, King Faisal Specialist Hospital and Research Centre, Riyadh, SAU; 13 Department of Infectious Diseases, Abdali Medical Center, Amman, JOR; 14 Pediatric Stem Cell Transplant Unit, Department of Pediatric Hematology/Oncology, National Bank of Kuwait (NBK) Children's Specialized Hospital, Sabah Central Health Region, KWT

**Keywords:** multidisciplinary, knowledge, therapeutic drug monitoring, resistance, antifungal use, antifungal stewardship

## Abstract

In recent years, global public health efforts have increasingly emphasized the critical role of antimicrobial stewardship (AMS) in improving outcomes, reducing costs, and combating the growing threat of antimicrobial resistance. However, antifungal stewardship (AFS) has remained relatively overlooked despite the staggering impact of invasive fungal infections (IFIs). This burden is particularly pronounced in hospitals worldwide, with the Middle East facing significant unmet needs. The rising population of immunocompromised individuals vulnerable to IFI has prompted an increased reliance on antifungal agents for both prevention and treatment. Given the considerable mortality associated with IFIs and the emergence of antifungal resistance, implementing AFS programs in hospital settings is becoming increasingly urgent. In this article, we offer expert insights into the strategies that can be used for successful antifungal stewardship program implementation in IFI. Drawing upon the extensive clinical experience of a multinational and multidisciplinary panel, we present recommendations for optimizing AFS practices. We delve into the challenges and practical considerations of tailoring local AFS initiatives to the evolving landscape of fungal infections. Additionally, we provide actionable recommendations and position statements for the effective implementation of AFS programs, informed by the collective clinical experiences of panel members across their respective countries of practice.

## Introduction and background

The global threat of antimicrobial resistance stands as a daunting challenge, and effectively treating infectious diseases remains crucial for determining health outcomes. In response to this escalating challenge, various stewardship programs have been seamlessly integrated into the comprehensive framework of healthcare delivery strategy [1]. While the global community has placed a growing emphasis on the significance of antibiotic stewardship to combat the rise of the growing antibiotic resistance threat in the past few years, antifungal stewardship (AFS) has garnered comparatively less attention [2].

While fungal infections may not be as prevalent as bacterial infections, fungal infections impose a substantial threat to global health, causing a significant burden of morbidity and mortality on hundreds of thousands of at-risk patients, leading to significant direct and associated healthcare costs. Fungal pathogens are accountable for over 13 million infections each year worldwide [3]. Invasive fungal infections (IFI) occur with an estimated annual incidence of 6.5 million cases, resulting in 3.8 million deaths, approximately 2.5 million of which are directly attributable to these infections [4]. This burden is notable in hospitals globally, with a particular emphasis on the Middle East, where significant unmet needs exist. These difficulties encompass insufficient recognition of the clinical manifestations of the illness, a lack of awareness about fungal infections, inadequate diagnostic methods, delays in commencing active therapy, restricted use of rapid diagnostic tests, less-than-optimal antifungal treatment, and a lack of comprehensive AFS implementation [5]. Excessive prescription of antifungal medications heightens the risk of adverse effects and drug interactions for patients while also fostering the development of resistant fungal strains. Moreover, antifungal agents rank among the most expensive antimicrobial drugs in hospital inventories, and the populations vulnerable to invasive fungal diseases (IFDs) are expanding [2]. These factors collectively contribute to elevated mortality rates and extended hospital stays among affected populations.

Creating stewardship initiatives that integrate fundamental principles, often referred to as core elements, is widely recognized as a key strategy for enhancing the appropriate use of antibiotics and antifungals [6]. While the principles of antimicrobial stewardship (AMS) are crucial for AFS, significant distinctions exist in terms of the patient demographics vulnerable to IFDs and diagnostic methodologies [7].

In the Middle East and Arab countries, there is a scarcity of data on the epidemiology of IFIs and antifungal susceptibility [8]. Previous studies have emphasized the importance of implementing antimicrobial stewardship and infection prevention and control programs in Arab countries to mitigate the potential emergence of resistance in this region [9].

Given the significant mortality associated with IFIs and the emergence of antifungal resistance, establishing AFS programs in hospitals becomes imperative. These programs play a crucial role in monitoring and advocating optimal practices in antifungal prescribing to address these multifaceted challenges.

This document provides a brief review of the general principles of AFS program implementation, highlights the specific challenges encountered in the Middle East, and offers recommendations for the successful implementation of AFS programs in managing IFIs in this region.

## Review

Consensus objectives and methodology

A multinational and multidisciplinary group of eleven key opinion leaders convened in Dubai, United Arab Emirates. The collaboration brings together professionals from different countries (Saudi Arabia, UAE, Iraq, Bahrain, Jordan, and Kuwait), which adds a multinational dimension to their expertise. Moreover, the group consists of individuals with diverse specialties such as infectious diseases, adult and pediatric hematology/oncology, and critical care, reflecting a multidisciplinary approach to tackling fungal infections. The authors are recognized as key opinion leaders in their respective fields, indicating that they possess significant experience, expertise, and influence in managing fungal infections. Their involvement in this collaboration suggests a concerted effort to leverage their collective knowledge for the advancement of understanding and management of fungal diseases. The authors hail from various prestigious institutions across the Middle East, each specializing in different aspects of healthcare delivery. Their diverse affiliations highlight the breadth of expertise brought to the collaboration, encompassing academic, clinical, and research settings. By bringing together experts from diverse backgrounds, the collaboration aims to provide comprehensive insights into the prevention, diagnosis, and management of fungal infections. This multidimensional perspective is crucial for addressing the complex challenges associated with these infections effectively.

The meeting’s objectives were to explore the challenges and practicality of having local AFS guided by changes in the fungal infection landscape and to create recommendations and position statements for implementing a successful AFS program based on the collective clinical experiences of the panel members in their respective countries of practice.

A survey comprising a series of questions addressing all aspects of successful AFS was distributed to the experts before the meeting to facilitate discussion. The survey responses served as the foundation for discussions during the meeting. Guided by an experienced moderator, the discussion integrated the panel's clinical insights with a comprehensive review of published literature.

The questions that guided the group discussion are highlighted in Table 1.

**Table 1:** Questions that guided the group discussion

What do you consider to be the fundamental recommendations or essential components of a successful AFS program when it comes to managing IFIs
What are the most significant gaps or areas of improvement when it comes to establishing and maintaining an up-to-date AFS program for managing IFIs?
What are the most significant clinical considerations or patient risk factors when using antifungal agents in managing IFIs? Are there specific factors that influence your choice of antifungal agents for different types of IFIs or different patient populations?
Do you have any recommendations regarding the appropriate timing for initiating antifungal therapy in suspected IFIs? Are there specific patient risk factors or clinical indicators that should influence this decision?
De-escalation and duration of therapy are critical aspects of AFS. How do you determine when to de-escalate or stop antifungal treatment for IFIs, and what factors guide these decisions?
How crucial are diagnostic methods in AFS, particularly in early detection and targeted therapy for IFIs? What are the available diagnostic tools & to what extent are they satisfactory during IFI management?
What role should TDM play in AFS for IFIs, and which antifungal agents require TDM?
Antifungal resistance is a growing concern. How do you recommend monitoring and responding to antifungal resistance patterns in the hospital or local community as part of stewardship efforts?
Are the protocols implemented in your hospital/center based on patients' records/databases or international guidelines & evidence?

Consensus was achieved on various topics related to AFS, including its importance, identified gaps, areas of improvement, drug monitoring, and resistance. These conclusions were drawn based on the specific challenges encountered in the region, as highlighted by the panel's collective expertise.

Review and expert opinion

Despite the increasing reliance on antifungal therapy, inappropriate utilization remains prevalent in hospital settings, contributing to drug resistance, elevated costs, and adverse events [2]. Limited access to fungal pathogen detection and antifungal susceptibility testing exacerbates these challenges, highlighting the urgent need for improved antifungal stewardship initiatives. Therefore, our study centers on identifying effective strategies for optimizing antifungal prescription practices, enhancing diagnostic capabilities, and overcoming implementation barriers to promote judicious antifungal use and mitigate the growing threat of resistance.

The rising population of immunocompromised individuals vulnerable to IFI has prompted an increased reliance on antifungal agents for both prevention and treatment. Many clinicians opt for early empiric antifungal therapy (EAFT) rather than awaiting confirmed diagnoses. Studies have indicated that delaying the treatment of candidemia is linked to increased mortality. A comprehensive systematic review conducted by Kanj et al. analyzed 16 articles investigating the impact of EAFT on the survival of patients with IFI, of which six reported that EAFT outperformed diagnostic-based treatment. This suggests that the timely initiation of EAFT, when deemed appropriate, has the potential to enhance patients' outcomes [10].

The majority of fungal infections are typically diagnosed as secondary conditions related to other medical issues rather than being the primary cause of hospital admission [3]. In the hospital setting, up to 50% of antifungals are utilized inappropriately [2,11]. Factors contributing to such inappropriate usage encompass improper prescriptions due to the use of incorrect administration routes, inappropriate dosages and incorrect indications for drug usage, inadequate treatment durations, and improper antifungal selection. Lack of microbiological adjustments based on local susceptibility patterns and the failure to discontinue treatment when the risk factors for IFI subside or when sepsis is determined to be caused by a different type of infection are also contributing causes [11]. Consequently, there has been a notable surge in antifungal drug resistance, namely among *Candida* and *Aspergillus spp.*, leading to higher costs and expenses, along with elevated rates of adverse drug events and drug-drug interactions [12]. This highlights a concerning pattern of suboptimal antifungal utilization, emphasizing the need for improved prescription practices in managing fungal infections.

Moreover, the routine availability of fungal pathogen detection and antifungal susceptibility testing in diagnostic laboratories is often either too time-consuming, non-specific, or lacking. For example, diagnosing invasive aspergillosis microbiologically depends on isolating *Aspergillus spp.* in culture, examining the samples under a microscope, and detecting the galactomannan antigen in serum and BAL fluid [13]. Despite their utility, these methods may have lengthy processing times and low specificity, especially in high-risk patients who are already on antifungal prophylaxis. Additionally, there exists a significant discrepancy in the availability of fungal biomarkers like galactomannan and beta-d-glucan both globally and within Middle Eastern territories. Numerous facilities throughout the Middle East lack direct access to these tests, necessitating the sending of samples to external reference laboratories. Consequently, this process results in a delay in obtaining results, rendering them clinically ineffective. In addition, *Aspergillus spp.* is seldom detected in blood samples, as evidenced by a study revealing that merely 6% of patients diagnosed with invasive aspergillosis had positive blood cultures [13]. This highlights yet another challenge in the laboratory detection of fungal species, emphasizing the importance of implementing AFS initiatives to address this effectively.

AFS centers on optimizing patient outcomes, simultaneously reducing healthcare costs, and safeguarding the future efficacy of the limited array of available treatment options [14]. However, the pace of new antifungals entering the market lags behind the rising trend of drug resistance. For example, amphotericin B remains the most potent fungicidal therapy in laboratory settings, exhibiting broad antifungal activity and often being employed to treat various systemic infections. However, due to its toxicity and side effects, it is typically reserved for severe infections in critically ill or immunocompromised patients. Newly approved triazoles, such as isavuconazole and posaconazole, offer enhanced fungicidal activity against Aspergillus. Nonetheless, isavuconazole is not approved for empiric *Candida* coverage due to the rising prevalence of azole-resistant infections caused by non-Albicans species [15]. This trend of antifungal resistance has been linked to rising healthcare costs, adverse drug reactions resulting from the need for increased dosages or combination therapy, an increased length of hospital stay, and heightened mortality rates. Therefore, AFS should align its objectives with antimicrobial stewardship (AMS) for judicious and sustainable usage of antifungal and antibiotic agents. Integrating antifungal stewardship within antimicrobial stewardship programs involves implementing strategies to optimize the use of antifungal agents alongside antibiotics. This includes educating healthcare professionals on antifungal stewardship principles, fostering multidisciplinary collaboration to develop consensus guidelines, and implementing formulary management strategies. Diagnostic stewardship is emphasized to ensure accurate diagnosis of fungal infections while monitoring and surveillance systems track prescribing practices and resistance patterns.

In implementing AFS strategies, a complex landscape of challenges emerges, necessitating thoughtful consideration for successful augmentation and transformation. Among these challenges are the imperative to enhance organizational structures, ensuring that education initiatives permeate broadly, and advancing microbiological diagnostic methods. Additionally, there is a critical need to elucidate the patient benefits associated with empirical antifungal therapy and establish effective strategies for its timely cessation. The refinement of de-escalation approaches for patients with confirmed infections and a comprehensive understanding of the advantages derived from therapeutic drug monitoring (TDM) further contribute to the intricate tapestry of challenges faced in the pursuit of successful AFS program implementation [16]. Overcoming these challenges and prioritizing research in these areas is crucial for effectively implementing antifungal stewardship programs.

Essential components of a successful antifungal stewardship program in managing invasive fungal infections, gaps, and areas of improvement

Harnessing Senior Leadership and Strategic Planning

The establishment of an effective AFS program hinges on strategic planning and strong senior leadership, as evidenced by existing literature. The fundamental recommendations underscore the importance of a sequential approach, beginning with raising awareness about the significance of IFI, the necessity for robust diagnostic infrastructure, and heightened suspicion of fungal infections [17]. This task is contingent upon the presence of senior leadership that will drive the AFS, allocate adequate resources, instigate necessary changes, and promote the integration of the core elements of the AFS. Numerous studies have indicated that leadership is recognized as a fundamental element in ensuring a well-coordinated and integrated provision of care, and thus, having solid senior leadership could prove to be an effective strategy for enhancing AMS. A study by Steinmann et al. showed that the change in leadership style, transitioning from a controlling approach to one focused on empowering frontline physicians, had a profound impact on the management of invasive fungal infections within the facility. The new leadership prioritized supporting and empowering frontline physicians, allowing them greater autonomy in decision-making within interdisciplinary teams. This shift led to the implementation of initiatives like an early extubation policy and streamlined antibiotic therapy guidelines. The empowering leadership style not only enabled frontline physicians to take direct responsibility for patient care but also fostered a sense of purpose and unity among team members, resulting in a heightened commitment to achieving self-defined goals. Importantly, this leadership approach was instrumental in driving improvements in antimicrobial stewardship, particularly in antibiotic use and reducing hospital-acquired infections [18]. Numerous studies have delved into the realm of effective leadership in health services over recent decades, prompted by societal challenges highlighting the crucial role of leadership in healthcare quality. Leadership was identified as pivotal for well-coordinated care provision, irrespective of care settings. Specifically, transformational and resonant leadership styles correlated with lower patient mortality, while relational and task-oriented leadership were linked to higher patient satisfaction [19]. Future research should focus on developing and implementing robust leadership models across diverse healthcare settings, incorporating multidisciplinary teams, and prioritizing the engagement of non-medical clinical leaders to ensure legitimacy and validity in priority-setting processes.

Following this, the next step involves quantifying the extent of the problem in terms of financial impact and life-saving significance. Subsequently, discussions should revolve around prevention strategies, followed by addressing specific cases and therapeutic approaches. This process entails evaluating available options, determining optimal choices, and establishing comprehensive guidelines.

Elevating Prescriber Education and Collaboration: Addressing Knowledge Gaps for Enhanced Patient Care

Emphasizing the education of prescribers, personnel training, supervision, and implementation of recommendations are critical elements in fostering expertise and ensuring adherence to clear guidelines and institutional care pathways. It is essential to employ collaborative strategies to ensure the engagement of the key practitioners who most frequently manage IFIs. In a cross-sectional multicenter survey-based study involving five European tertiary care hospitals, Valerio et al. revealed a significant lack of knowledge among European physicians working in areas with the highest antifungal drug consumption. The study identified gaps in various aspects, such as distinguishing colonization from infection, antifungal prophylaxis indications, and pre-emptive therapy recommendations [20]. A similar study conducted in Saudi Arabia by Ibrahim et al. highlighted comparable deficiencies in the knowledge and practice of clinicians prescribing antifungal therapy in the region. Specifically, challenges were observed in areas related to treatment de-escalation, accurate identification of the appropriate antifungal treatment, and differentiation between *Candida* urinary infection and colonization [21]. Healthcare professionals face significant difficulties when managing patients with IFI due to their limited knowledge of symptoms, diagnostic techniques, and the proper usage and dosing of antifungal drugs [22]. Therefore, we propose the establishment of focused educational initiatives within a comprehensive AFS program, aiming to bridge knowledge gaps related to the interpretation of microbiology laboratory findings, distinguishing between colonization and infection, determining indications for prophylaxis versus empiric therapy, and understanding dosing and monitoring aspects of antifungal therapy. Implementing an effective AFS program presents more complexities compared to antimicrobial initiatives, as managing antifungals proves even more challenging due to factors such as raising awareness regarding both the presentation and appropriate timing for suspecting fungal infections among experts who treat them at local levels. In Middle Eastern countries, the majority of clinicians adhere to international guidelines for the treatment of invasive fungal infections when local or regional protocols are lacking [22]. Some hospitals have developed their own local protocols, drawing from these international guidelines.

Advancing Diagnostic Precision for Enhanced Treatment Outcomes

The next important step in AFS is accurate diagnosis and treatment. The timely detection and initiation of effective antifungal treatment are directly associated with enhanced survival in cases of fungal diseases [23]. There is a clear need for accurate pathogen identification and obtaining antifungal susceptibility testing whenever feasible for tailored and effective treatment, which may even support the step-down transition to oral therapy [2]. This will allow for the employment of the smallest effective-spectrum antifungal to minimize side effects and enhance treatment precision, as well as TDM. Effective pathogen identification requires specialized laboratories, expert technicians, and dedicated resources. Mycology laboratories still lag behind microbiology labs in terms of expertise and financial support since they are often considered part of microbiology labs instead of being recognized separately. Specialized mycologists must be incorporated into separate facilities offering necessary diagnostic tests where they are always available [24]; otherwise, relying on empiric antifungal treatment becomes essential under certain circumstances. Diagnostic challenges, such as a deficiency in fungal diagnostic testing compared to bacterial and viral testing, a lack of rapid diagnostic tools, and an absence of susceptibility testing, underscore a significant gap in fungal stewardship. This gap impedes the timely and accurate identification of fungal infections, contributing to delays in appropriate treatment and potentially leading to suboptimal patient outcomes. An expert opinion from the Fungal Diagnostics Laboratories Consortium identified several diagnostic gaps in the laboratory diagnosis of fungal diseases, encompassing the absence of molecular detection methods for fungal diseases and a deficiency in optimal diagnostic algorithms integrating fungal biomarkers and molecular tools for precise diagnosis of IFI [13]. There is an imperative need to embrace newer diagnostic tests for the accurate and timely diagnosis of IFI. Traditional diagnostic methods, although valuable, often lack specificity, are time-consuming, and can yield inconclusive results [13]. Enhancing fungal diagnostics requires diverse diagnostic tools, encompassing Matrix-Assisted Laser Desorption-Ionisation-Time of Flight Mass Spectrometry (MALTI-TOF MS), fungal biomarkers, antigen and antibody testing, molecular tests, and nucleic acid amplification tests. Relying on a single technology is insufficient for comprehensive improvement in fungal diagnostics [13,24]. The adoption of newer diagnostic tests holds promise for enhancing the efficiency and accuracy of IFI diagnosis, particularly in settings where conventional methods may be limited or inadequate. The turnaround time for fungal testing holds significant clinical importance, especially in critically ill or immunocompromised patients, where delays can significantly impact outcomes. Rapid identification of the fungal pathogen allows for targeted treatment, optimizing efficacy and reducing the risk of morbidity and mortality associated with IFIs. 

Multidisciplinary Collaboration

The success of an AFS program hinges on the functionality of a well-coordinated multidisciplinary team. Nurses, pharmacists, physicians, and all caregivers should incorporate the intricate aspects of antifungal management. In a study conducted by Nivoix et al., they assessed all systemic antifungal prescriptions across three departments (oncology and hematology, medical intensive care, and surgical intensive care), encompassing 70% of all systemic antifungal agent prescriptions in a tertiary care university hospital. Unlike other evaluations of antifungal therapy, their assessment was exhaustive, considering factors such as the indication, appropriateness of loading and maintenance doses, adjustments for renal and hepatic functions, adaptation to mycological results, and analysis of potentially dangerous drug-drug interactions [25]. However, the results, indicating a mere 34% rate of appropriate prescriptions, underscore the significance of a multidisciplinary team of experts. This team should include pharmacists to ensure proper drug dosing, conduct TDM, and avoid drug-drug interactions. Microbiologists play a crucial role in accurately directing therapy through the early identification of IFI and understanding the accessibility, performance, and interpretation of the available mycological tests. Infectious disease specialists and clinical microbiologists are essential for implementing recommendations and guidelines and providing clinical diagnosis of IFI. Additionally, the involvement of hematologists and intensive care specialists is vital for addressing the specific needs of immunocompromised patient populations [26]. Given the intricate nature of diagnosing and treating IFI, especially in critically ill patients or in patients undergoing chemotherapy, it is crucial to establish a collaborative multidisciplinary team to ensure a diverse approach to enhance patient care, minimize risks, and avoid drug-drug interactions, especially in patients who are often on polypharmacy [21,27].

Navigating the Challenges of Defining Clinical Diseases: Opportunities for Innovation and Consistency

As with any program implementation, ongoing monitoring and effective surveillance of fungal infections and antifungal use are essential [28]. This includes evaluating treatment outcomes and assessing the efficacy of the AFS program through indicators such as mortality rates, morbidity rates, resistance rates, and financial costs.

A primary reason for our challenges in achieving success with AFS programs, compared to AMS, is the absence of a standardized definition for clinical diseases. There is no universal agreement on defining these diseases, leading to variations in definitions across European, American, and local societies. This lack of consistency creates a significant opportunity for innovation in new approaches to prescriptions and treatments. In an effort to update the definitions of invasive fungal disease, several meetings have been held, where the categories of 'probable' and 'possible' disease have been slightly expanded, and indirect assays specific to the infection being detected have been added in the 'proven' group [29]. However, failing to meet the criteria for IFD does not imply the absence of IFD; rather, it indicates inadequate evidence to support the diagnosis. In a paper by Bassetti et al., the authors attempted to propose definitions for IFI in the Intensive Care Unit (ICU) setting based on the EORTC/MSGERC definitions of IFDs. The authors, however, concluded that there are challenges in creating IFD definitions that can be applicable in the ICU setting, owing to the variability of predisposing factors and the unreliability of alternative definitions as the reference standard for evaluating tests [30].

Enhancing Research and Collaboration in Fungal Infection Epidemiology

There exists a widespread deficiency in conducting large-scale burden of disease studies, and simultaneously, there is a significant lack of communication among various stakeholders. This lack of comprehensive research and communication poses challenges to understanding the true extent of disease burdens and hinders collaborative efforts to address them effectively. Fungal infection research has received significantly less development funding and research investment than other infectious disease topics [31]. Existing treatments for fungal diseases are inadequate, and a substantial investment in specialized research is necessary to explore new therapeutic alternatives. It is crucial to foster increased research initiatives and establish robust channels of communication among stakeholders, fostering a more holistic and collaborative approach to tackling health challenges.

There is a crucial need to amplify support and funding for research and data generation, particularly in the realm of the epidemiology of fungal infections. This involves allocating national resources to facilitate comprehensive studies that delve into the prevalence, incidence, risk factors, and outcomes associated with fungal infections. Increased financial investment will empower researchers to conduct large-scale studies, implement advanced diagnostic techniques, and gather real-world data to inform evidence-based strategies. Fostering collaborations between research institutions, healthcare providers, and public health agencies can contribute to a more holistic approach to addressing the challenges posed by fungal infections and advancing the field of AFS.

Expert considerations for the use of Antifungal Agents

Navigating Complexities in Patient Characteristics, Disease Characteristics, and Clinical Presentation

In the intricate landscape of managing IFIs, systematic consideration of patient, disease, and treatment characteristics is essential. When antifungal therapy is initiated, it is important to integrate multiple risk factors into risk scores in order to guide decision-making. For example, in patients with hematological diseases, a set of host-related factors should be taken into consideration, such as the type of underlying disease accounting for the diverse immunosuppressive states, the status of the disease, host fitness, age, and immunogenic status [32]. The authors suggest that patient characteristics, such as the immune status of the host (encompassing hematological malignancies, neutropenic patients, organ transplant recipients, and those undergoing chemotherapy), comorbidities (especially diabetes or renal impairment), age, and the presence of medical devices such as urinary catheters or implantable cardiac devices, play pivotal roles in shaping the treatment approach. The primary duty of the patient's physician is to deliver care promptly, seek consultations, and determine specific procedures, drug selection, and dosage based on the patient's most up-to-date information. These decisions should be carefully weighed, considering all pertinent clinical findings and the patient's best interests.

Further delving into disease characteristics is imperative for a comprehensive understanding of the factors influencing the management of fungal diseases. The location and severity of the fungal disease play pivotal roles in determining the appropriate therapeutic approach, considering the potential impact on surrounding tissues and organs. Additionally, discerning the specific type of infection is crucial for tailoring treatment plans to address the unique challenges posed by different fungal pathogens [32]. The clinical presentation serves as a valuable diagnostic clue, offering insights into the manifestation of symptoms and the patient's overall health status. It guides healthcare providers in assessing the urgency and intensity of the intervention required. Adherence to hospital prophylaxis guidelines becomes paramount in preventing the onset or progression of fungal infections, especially in vulnerable patient populations. Moreover, the availability of advanced diagnostic tools and susceptibility testing enhances precision in decision-making. Timely and accurate diagnostics contribute to the swift identification of the fungal species involved, enabling a targeted and effective treatment strategy. Susceptibility testing assists in determining the most suitable antifungal agent, considering the unique characteristics of the identified pathogen and its potential resistance to certain drugs.

Strategies for Initiating Antifungal Therapy in Suspected Invasive Fungal Infections

Initiating antifungal therapy in suspected IFIs should prioritize early intervention, taking into account several key considerations. The decision to commence antifungal treatment should be guided by the patient's risk category, clinical suspicion of IFI, individual risk factors, and a comprehensive clinical assessment. Prophylactic treatment involves administering an antifungal agent preventively to individuals at risk of IFIs who do not exhibit attributable signs and symptoms. Empiric treatment, on the other hand, entails initiating antifungal therapy in high-risk patients displaying clinical signs and symptoms of IFIs but lacking microbiological confirmation. Pre-emptive therapy is implemented when the decision for treatment relies on early diagnostic tests. Lastly, targeted therapy requires the identification of the specific pathogen before being defined and applied [33]. In hospitals where rapid diagnostic tools are not readily available, initiating empiric treatment aligned with risk stratification is recommended, as per the authors of this paper. However, a pre-emptive approach may be considered in more advanced medical centers. Opting for a pre-emptive approach has the potential to reduce the number of patients undergoing treatment and significantly alleviate the financial burden associated with antifungals, all without surpassing the incidence rate of IFI [34,35]. A randomized trial comparing empiric vs. preemptive antifungal strategy in high-risk neutropenic patients on fluconazole prophylaxis has shown that a preemptive antifungal approach comprising biweekly galactomannan screening and CT scans as needed does not negatively impact the overall survival of adult patients experiencing prolonged neutropenia and at high risk for invasive fungal disease while on fluconazole prophylaxis. Furthermore, this strategy does not elevate the likelihood of a proven or probable IFD. Notably, it results in a 50% reduction in antifungal usage, suggesting potential cost savings [34]. This alleviates the burden of excessive antifungal use without increasing mortality or increasing the incidence of IFI.

Considerations for De-Escalation of Antifungal Therapy

Key factors influencing the decision to de-escalate from a broad to a narrower-spectrum drug or to discontinue antifungal treatment for IFIs are susceptibility results, the patient's clinical improvement, and resolution of symptoms, indicating a positive response to the therapy, or investigation results necessitating drug cessation. Reasons for de-escalation include susceptible strains based on antifungal susceptibility testing or culture conversions to negative. Studies have shown that even in the ICU setting, antifungal de-escalation did not exhibit any adverse effects on the duration of mechanical ventilation, length of stay in the ICU, ICU mortality, 28-day mortality, or one-year mortality rates [36,37]. The evaluation of treatment duration is meticulous, considering the minimum required for effective management. At the same time, the assessment of clinical progression and the specific fungal infection type inform the determination of the appropriate duration. Risk assessment involves considering host factors and integrating rapid diagnostics, biomarkers, and cultures. The clinical response is carefully evaluated in conjunction with these risk factors. The exploration of alternative diagnoses may warrant the discontinuation or de-escalation of antifungal therapy. Other clinical indications for such decisions include the recovery of neutropenia in immunosuppressed patients, subsiding fever, and improvement in imaging studies. Negative results from microbiological samples, including blood cultures, bronchoalveolar lavage samples, and galactomannan tests, play a crucial role in the decision-making process. The likelihood of a fungal infection influences the ultimate choice to de-escalate or cease antifungal therapy. The involvement of an infectious disease specialist is essential to ensuring a comprehensive and expert-driven decision-making de-escalation process.

Therapeutic Drug Monitoring Enhances Antifungal Treatment Precision

Therapeutic drug monitoring (TDM) is pivotal in the realm of AFS, serving a crucial role in optimizing efficacy, averting toxicity, and mitigating the risk of drug interactions [38]. Integrating TDM into AFS programs goes beyond a standardized approach, contributing significantly to individualized patient care. This becomes particularly valuable for patients with factors influencing drug metabolism, including conditions like liver dysfunction or those susceptible to drug interactions. By tailoring antifungal therapy based on TDM results, healthcare professionals can navigate complexities related to patient-specific factors, ensuring a more precise and effective treatment strategy. Azoles such as Voriconazole, Itraconazole, and Posaconazole derive substantial benefits from TDM owing to their unpredictable pharmacokinetics and narrow therapeutic indices [39]. This holds particular relevance when considering ethnic variations that impact azole metabolism, especially in regions like ours with a significant population of Asian descent. Implementing TDM becomes crucial in ensuring optimal dosing and therapeutic outcomes for individuals undergoing azole therapy, addressing the challenges posed by inter-individual variability in drug metabolism [40]. Medical practitioners often overlook the fact that the therapeutic concentration ranges provided by reference laboratories are based on diverse patient populations. What may be considered an appropriate therapeutic target for one patient might not necessarily be suitable for another. As a result, TDM necessitates ongoing clinical involvement to ascertain that suitable targets are selected, avoiding a generic 'one size fits all' approach [39]. The utilization of TDM predominantly relies on clinical judgment and the unique characteristics of individual patients. TDM is particularly warranted for patients exhibiting unpredictable pharmacokinetics, such as obese or critically ill patients; pharmacokinetic variabilities, such as those with severe diarrhea or those switching from IV to oral; or those concurrently using medications that may either decrease or increase the concentrations of antifungal agents. TDM is particularly advised for most individuals prescribed voriconazole, posaconazole, itraconazole, and flucytosine [41]. Currently, there is no evidence or indication supporting the routine application of TDM for polyenes (such as amphotericin B deoxycholate, liposomal amphotericin B, and amphotericin B lipid complex) or the echinocandins (including micafungin, caspofungin, and anidulafungin) [39].

Balancing Accessibility and Efficiency

Various methods, such as bioassay, high-performance liquid chromatography, and mass spectrometry, have been employed to measure serum concentrations of antifungal agents [42]. However, the turnaround time is an additional crucial consideration in the context of TDM. While having on-site assays may be ideal, the associated costs of developing and running these assays often limit the availability of TDM services to specialized centers. This limitation can lead physicians to avoid certain drugs if TDM is not readily accessible. Even when TDM services are available, the efficiency of the turnaround time becomes crucial. Timely availability of results is imperative for physicians to make informed decisions promptly. Therefore, the focus should not only be on the mere availability of TDM services but also on the efficiency and promptness of the turnaround time, ensuring that the results are accessible to physicians promptly.

In certain situations, physicians may avoid prescribing a drug if TDM is unavailable. However, the experts propose another perspective, which emphasizes an all-or-none approach when using azoles for critically ill patients, suggesting that TDM is essential to assess treatment success or failure and determine whether the failure is due to medication availability at tissue concentration, patient condition, or breakthrough infections. Therefore, in tertiary care centers where these medications are frequently required, incorporating TDM should be considered a standard of care.

Monitoring and Responding to Antifungal Resistance Patterns

Antifungal resistance is now an emerging concern that is recognized as a threat to public health. The development of antifungal resistance typically stems from alterations that impact the interaction between the drug and its target, either directly or indirectly. Resistance can emerge through genetic modifications affecting the binding site of the target, via overexpression of the availability of the target, or by modification of effective drug concentration (such as heightened drug efflux activity for intracellular drugs like azoles, or inhibition of prodrug activation as observed with flucytosine) [43,44]. A comprehensive approach is crucial to effectively monitoring and responding to the escalating concern of antifungal resistance in hospitals and local communities. Implementing infection control isolation precautions is imperative to curb the spread of resistant strains.

Utilizing Epidemiological and Laboratory Data for Informed Response Strategies

Leveraging epidemiological and laboratory data allows for continuous monitoring of resistance patterns, facilitating a prompt response. The development of cumulative antifungal susceptibility reports contributes to a comprehensive understanding of the evolving resistance landscape. Due to the labor-intensive and complex nature of the 'gold standard' reference techniques for Antifungal Susceptibility Testing, numerous clinical laboratories opt for commercial tests. While these tests are convenient, they may not be entirely standardized across all drug-fungus combinations, leading to potential misclassification of susceptibility results compared to the reference tests [45]. Ensuring unified breakpoints for sensitivity testing maintains consistency across testing for all invasive isolates.

Given that numerous routine diagnostic laboratories, particularly in lower and middle-income settings, are often unable to conduct routine species identification, clinicians worldwide must have access to local epidemiological data and susceptibility patterns from reference laboratories. This access helps guide treatment choices more effectively. Considering alternative antifungal agents guided by susceptibility testing results ensures effective response strategies.

Local fungal antibiograms play a pivotal role, providing insights into regional resistance profiles. Empirical use should be guided by these antibiograms to align treatment strategies with local resistance patterns. Collaborating with infectious disease specialists offers expert guidance, enhancing the precision of stewardship efforts. Multidisciplinary communication is essential for continuous monitoring, involving specialists and departments in the exchange of information. It also facilitates the establishment and monitoring of outcome metrics related to antifungal susceptibility patterns. By collaborating across specialties, healthcare teams can define relevant metrics, such as rates of antifungal resistance, clinical response to therapy, and patient outcomes. Regular communication enables the tracking of these metrics over time, allowing for the identification of trends and areas for improvement in antifungal stewardship practices. Furthermore, the exchange of information regarding outcome metrics fosters accountability and transparency among healthcare professionals, driving continuous quality improvement efforts aimed at optimizing patient care and mitigating the impact of antifungal resistance.

Discussion

The results provided in this expert opinion paper highlight several critical components essential for the successful implementation of antifungal stewardship (AFS) programs in managing invasive fungal infections (IFIs). These components encompass strategic planning, prescriber education, diagnostic precision, multidisciplinary collaboration, continuous monitoring, clinical considerations, therapeutic drug monitoring (TDM), and response strategies to antifungal resistance. Advocacy for national programs, such as those incorporating specialized microbiologists and comprehensive surveillance initiatives, is vital [10]. Adaptive stewardship policies should be regularly adjusted based on emerging resistance patterns. Educational initiatives targeting healthcare professionals are crucial for raising awareness and promoting adherence to updated protocols, fostering a collective effort against antifungal resistance. This multifaceted approach, incorporating local and national elements, strengthens stewardship efforts and systematically enhances the ability to address antifungal resistance.

The panelists identified the following key considerations (Table 2):

**Table 2:** Key considerations identified

There exists an unmet need for unified recommendations concerning fungal infections and antifungal therapy.
AFS initiatives play a crucial role within the framework of all-inclusive stewardship programs in any hospital/center treating fungal infections or utilizing antifungal therapy
The current consensus serves as general recommendations for practitioners and stakeholders. However, it should not substitute each physician's experience and should be tailored to distinct patients, accounting for regional and institutional variations.
The recommendations reported in this consensus may not apply to specific populations such as immunocompromised patients.

The strength of this study lies in its comprehensive approach, which integrates insights from several key opinion leaders and experts in the field of antifungal stewardship. This diverse representation of experts in infectious diseases, hematology/oncology, and critical care from multiple countries ensures a thorough exploration of the challenges and practicalities related to antifungal stewardship across different healthcare contexts. The insights derived from real-world clinical practice enhance the credibility and relevance of the recommendations and position statements generated by the panel members. The study employs a structured methodology, including the distribution of a survey addressing all aspects of successful AFS, guided group discussions based on survey responses, and a comprehensive review of published literature. This systematic approach ensures a thorough exploration of key topics and facilitates consensus-building among the panel members.

However, limitations exist within the study framework. While the study draws insights from a multinational panel, its focus on the Middle East region may limit the generalizability of the findings to other geographical areas with different healthcare systems, epidemiological profiles, and resource availability. The unique challenges and practices specific to the Middle East region may not fully reflect the broader global landscape of AFS. Despite efforts to ensure diverse representation, the perspectives and recommendations generated by the panel members from six Middle Eastern countries may still be influenced by regional biases or local practices prevalent in their respective countries of practice. This could affect the applicability of the study findings to regions within and outside the Middle East. Furthermore, the study primarily involves input from key opinion leaders, potentially overlooking perspectives from other stakeholders, such as patients, healthcare administrators, and policymakers. Involving a broader range of stakeholders could provide additional insights and enhance the relevance and impact of the study findings.

## Conclusions

This document explores the challenges and practical considerations of implementing local AFS initiatives. It provides insights into developing effective strategies for successful AFS program implementation, especially in IFI and antifungal therapy. Addressing prevalent issues such as inappropriate antifungal use, the growing public health concern of antifungal resistance, and the absence of a fundamental framework, the establishment of AFS programs in each institution represents a significant stride toward improving patient care in IFIs. While many of these strategies can be globally applicable, tailoring them at the local level is essential due to variations in healthcare systems and practices. These programs foster improved communication, diagnosis, and management and contribute to optimizing patient outcomes and increasing cost-effectiveness.
